# Conversion of plastic waste into fuel oil using zeolite catalysts in a bench-scale pyrolysis reactor†

**DOI:** 10.1039/d1ra08673a

**Published:** 2022-03-08

**Authors:** Krishnasamy Sivagami, Keshav V. Kumar, Perumal Tamizhdurai, Dhivakar Govindarajan, Madhiyazhagan Kumar, Indumathi Nambi

**Affiliations:** Environmental and Water Resources Division, Department of Civil Engineering, Indian Institute of Technology Madras Chennai-600 036 India indunambi23@iitm.ac.in +91-44-2257-4289; Department of Chemistry, Dwaraka Doss Goverdhan Doss Vaishnav College (Autonomous) E.V.R. Periyar Road, Arumbakkam Chennai Tamil Nadu 600 106 India p.tamizhdurai@dgvaishnavcollege.edu.in +91-9677146579; Samudhyoga Waste Chakra Private Limited IIT Madras Research Park, Tharamani Chennai-600 113 India; Industrial Ecology Group, School of Chemical Engineering, Vellore Institute of Technology Vellore-632 014 Tamil Nadu India

## Abstract

Catalytic pyrolysis of mixed plastic waste to fuel oil experiment was tested with ZSM-5 zeolite (commercial and synthesized) catalysts along with other catalysts. The ZSM-5 zeolite catalyst was effectively produced using a hydrothermal technique *via* metakaolin as an alumina source. The catalytic pyrolysis of different types of plastic (single and multilayer) wastes in the presence of various catalysts was tested with a bench-scale pyrolysis setup with 2 kg per batch capacity. Polyolefin based plastics (low-density polyethylene, high-density polyethylene, and polypropylene), multilayer plastics such as biaxial oriented polypropylene (BOPP), metalized biaxial oriented polypropylene layers (MET BOPP), polyethylene terephthalate (PET), metalized polyethylene terephthalate (MET/PET), polyethylene terephthalate combined polyethylene (PET/PE), and mixed plastic waste collected from the corporation sorting center were pyrolyzed in a batch pyrolysis system with 1 kg feed to determine the oil, gas and char distributions. The performances of commercial ZSM-5 and lab synthesized ZSM-5 catalysts were compared for the pyrolysis of non-recyclable plastic wastes. Other commercial catalysts including mordenite and gamma alumina were also tested for pyrolysis experiments. The gross calorific value of oil obtained from different combinations of multilayer packaging waste varied between 10 789–7156 kcal kg^−1^. BOPP-based plastic waste gave higher oil yield and calorific value than PET-based plastic waste. Sulfur content present in the oil from different plastic wastes was measured below the detection limit. The synthesized ZSM-5 zeolite catalyst produced a maximum oil output of 70% and corresponding gas and char of 16% and 14% for LDPE plastic. The strong acidic properties and microporous crystalline structure of the synthesized ZSM-5 catalyst enables increased cracking and isomerization, leading to an increased breakup of larger molecules to smaller molecules forming more oil yield in the pyrolysis experiments. Residual char analysis showed the maximum percentage of carbon with heavy metal concentrations (mg kg^−1^) in the range of *viz.*, chromium (15.36–97.48), aluminium (1.03–2.54), cobalt (1.0–5.85), copper (115.37–213.59), lead (89.12–217.3), and nickel (21.05–175.41), respectively.

## Abstract 2

Catalytic pyrolysis of mixed plastic waste to fuel oil experiment was tested with ZSM-5 zeolite (commercial and synthesized) catalysts along with other catalysts.

## Graphical abstract

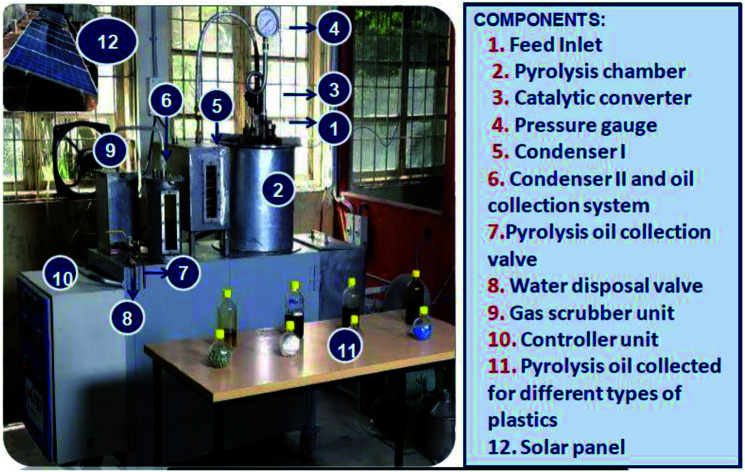

## 1. Introduction

The rate of generation of plastic waste is increasing exponentially. This is primarily because of the increased production of plastics and the low recycling rate around the globe. For instance, the production of plastics has increased from 250 million metric tons in 2008 to 335 million metric tons in 2016.^1^ However, only less than 10% of the total plastic waste generated is recycled, while the rest is found in landfills or oceans.^2^

The most commonly used areas of plastic in our daily lives are packaging, building and construction plastic, automotive, electrical and electronic, agriculture, and sports.^3^ The usage of plastic in these areas has been inevitable due to low price, the durability of plastic, prevention of food waste and contamination, and reduced weight of the packaging. On the downside, the non-degradable nature of plastic waste unbalances the ecosystem. About 5–13 million tons of plastic end up in the ocean every year.^2^ The municipal solid waste (MSW), which comprises 10–12% of plastic, is also burned, releasing toxic gases such as dioxins, furans, mercury, and polychlorinated biphenyls.^4^

Recycling plays a key role in ensuring these plastics do not reach the ocean or the landfill. Recycling is broadly classified into physical and chemical recycling.^5^ Physical recycling involves sorting, washing, cleaning, and shredding plastic waste to re-extrude plastic. The plastic waste undergoes oxidation, radiation, and heating in mechanical recycling, degrading the polymer quality.^6^ Additionally, it is challenging to treat mixed plastic waste through mechanical recycling due to the difference in melting point and processing temperature.

These disadvantages of mechanical recycling can be overcome with chemical recycling. Chemical recycling is a process of converting polymers to monomers through a thermochemical or catalytic process. Pyrolysis is a widely used chemical recycling process to convert different types of plastic waste to liquid fuel. Pyrolysis is a process of breaking down long-chain hydrocarbons into smaller chains with the application of heat and pressure in the absence of oxygen. The process is carried out in a wide range of temperatures from 300 to 900 °C. The process yields three products (1) liquid oils, (2) non-condensable gases, and (3) char (solid). Studies have shown oil recovery of up to 80% with gaseous and char byproducts.^7^ The gaseous byproduct with a high calorific value is used as a secondary heat source to reduce the overall energy requirement of the system.^8^ The application of liquid oil includes usage in boilers for combustion, engines, turbines, and chemical feedstock.^9^

The conventional pyrolysis systems are temperature-dependent, and the liquid fuel recovered from the process might contain residues and impurities.^10^ The low selectivity nature of the pyrolysis process often leads to uncontrolled product distribution.^11^ Additionally, the conduct of polyolefin-based plastics such as high-density polyethylene (HDPE), low-density polyethylene (LDPE), and polypropylene (PP) is difficult in temperature-dependent processes in the absence of catalysts due to the crossed chain hydrocarbon structures. Thus, it is of critical importance to use a catalyst to generate products in the range of commercial-grade fuels such as gasoline to ensure economic viability for pyrolysis plants.^12–15^

The usage of catalysts to improve product distribution and selectivity has been studied over the past two decades. A range of catalysts has been tested, such as commercial and domestic activated carbon, modified natural zeolite (NZ) catalyst,^16^ two-stage catalysis using mesoporous MCM-41 followed by microporous ZSM-5,^17^ Ni/Al_2_O_3_ catalyst,^18^ HZSM-5 zeolite, ZnO, silica, calcium carbide, alumina, magnesium oxide, zinc oxide and homogeneous mixture of silica and alumina,^19^ ZSM-5 zeolite and Red Mud.^20^ The usage of these catalysts improves the product distribution, reduces the temperature required for the process, and thus significantly reduces the energy consumption and ensures faster reaction time.^21^ Additionally, the high selectivity in catalytic pyrolysis holds a key advantage against the thermal degradation process by simulating isomerization.^22^

The catalytic processes can be broadly classified as homogeneous and heterogeneous processes. A homogenous catalyst is a single-phase catalyst, whereas a heterogeneous catalyst is a solid. Heterogeneous catalysts are widely used due to their ability to withstand extreme conditions such as temperatures up to 1300 °C and pressure of 35 MPa. Studies have shown acid–base catalysts (zeolite) to be more effective than less acidic catalysts (silica–alumina).^23^ Most of the above-mentioned studies have experimented with plastics such as LDPE, HDPE, and PP. However, as mentioned before, the real composition of plastic waste collected from municipal solid waste contains a lot of post-consumer packaging waste multi-layered plastic mixed with polyolefins.^24,25^

Furthermore, from an operational point of view, given that the capacity of plastic pyrolysis plants ranges from 5 tons to 50 tons per day, the cost structure of the plant and its return of investment is highly dependent on the consumption, utility, and manpower expenses.^26^ The major cost of consumables includes the amount of catalyst used and the price per kg of the catalyst.^27,28^ Hence, the unit economics of the plant and its profitability is dependent on the catalyst expense, among other factors. Studies conclude that the type and amount of catalyst are the key distinctions between thermal and catalytic pyrolysis, and any system would aim to use catalysts at practically zero cost.^29–31^ Thus, it is of importance to study and compare different catalytic pyrolysis systems that are more efficient and cheaper than the commercially available catalysts.^16,26^ To date, very few studies have been published comparing the use of these catalysts for real plastic waste and its impact on product distribution.^32–34^ Besides, these studies have been carried out with real plastic waste with a catalyst such as red mud and zeolite.^20^

The study of real plastic waste includes the following (a) real plastic wastes from the residue of a material recovery facility (MRF) that respond differently to pyrolysis than simulated samples, which is composed of just a few pure plastics (b) a combination of multilayered plastic (MLPs) with polyolefin-based mixed plastic waste. A real plastic study with a low-cost catalyst would lead to a feasible solution to obtain a consistent pyrolysis oil quality and quantity. Based on the above discussion, it is well known that the study of mixed plastic waste with different catalysts is quite limited. Therefore, the interest of this study lies in the pyrolysis of actual plastic solid wastes collected in India exhaustively with the synthesized low-cost catalyst and commercial catalysts.

## 2. Materials and methods

3-[(Trimethoxysilyl)propyl] octadecyldimethylammoniumchloride (ODAC, 60% methanol solution), metakaolin, and TEOS (tetraethyl orthosilicate) were purchased from Sigma Aldrich. Sodium hydroxide pellets (NaOH) were purchased from S. D. Fine Chemicals Ltd. Tetrapropyl ammonium bromide (TPABr) was purchased from Merck, India.

### 2.1. Preparation of metakaolin (metakaolinization)

The metakaolin phase is amorphous and highly reactive as compared to the kaolin phase and that phase is obtained by dehydroxylation of kaolin at a higher temperature. In this study, we used metakaolin as an alumina source and treated it at 600 °C for 1 h with a heating rate of 10 °C min^−1^ before using it.^35^

### 2.2. Synthesis of micro-mesoporous zeolites ZSM-5 catalyst

Micro-mesoporous ZSM-5 molecular sieves were synthesized hydrothermally from a reaction gel containing 3-[(trimethoxysilyl)propyl] octadecyldimethyl-ammoniumchloride (ODAC, 60% methanol solution) and tetrapropyl ammonium bromide (TPABr) as templates by following a modified recipe of previously reported procedures.^36^ A typical synthesis gel was prepared as follows: solution A was prepared by adding 0.4 g of NaOH pellets, followed by the addition of 1.4 g of TPABr and 0.5 g of metakaolin in 67.5 g of distilled water under vigorous stirring at room temperature for 30 min until the solution was homogeneous. Finally, a gel was obtained by adding a homogenous mixture of 4.285 g of TEOS (tetraethyl orthosilicate) and 0.595 g of ODAC into solution A under vigorous stirring for 2 h. Gels with chemical compositions containing different amounts of ODAC as represented by the formula, 1Al_2_O_3_ : 2.33TPABr : 2.22Na_2_O : 11.04–11.85SiO_2_ : 0.26–1.6ODAC : 1666H_2_O were prepared to obtain the different ZSM-5 samples. The mixture was then transferred into a stainless-steel autoclave heated in an oven at 150 °C for 24 h.^37^ The obtained crystallized product was washed, dried at 100 °C overnight, and calcined in air at 550 °C for 6 h with a heating rate of 1 °C per min. Other ZSM-5 samples were synthesized with different SiO_2_/Al_2_O_3_ ratios of 5, 10, 20, and 50 by varying the TPABr/ODAC ratios. The synthesized catalyst is used for pyrolysis of plastic solid waste (PSW) and performance comparison with industrial catalysts.^38^

### 2.3. Plastic waste sample collection

Plastic waste samples were collected from different sources. Polyolefin wastes like polyethylene, polypropylene, and shredded mixed plastic waste were collected from plastic recyclers and corporation material recovery facilities in Chennai. Plastic wastes were collected from flexible packaging industries in Chennai.

### 2.4. Batch plastic pyrolysis reactor

**Fig. 1:**
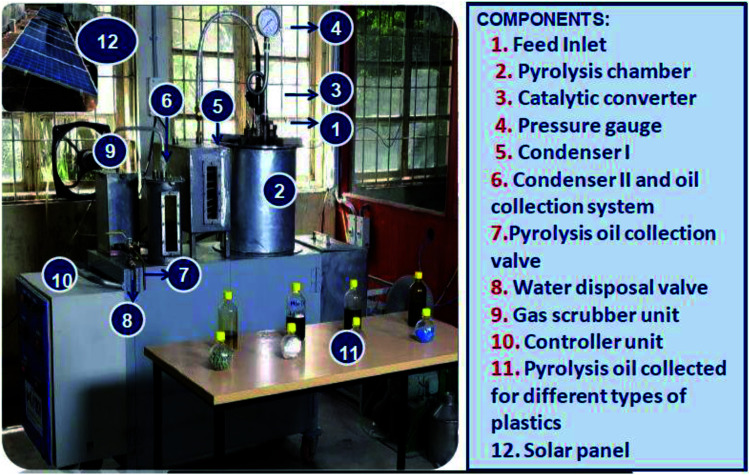
Photograph of plastic pyrolysis bench-scale plant.

Fig. 1 shows the photographic image of a plastic pyrolysis bench-scale plant. Batch pyrolysis experiments were carried out with 1 kg of PSW-based plastic waste and the required amount of catalysts like zeolite, ZSM-5 (commercial grade and synthesized), and mordenite. The PSW and the catalyst were fed into the inner chamber of the pyrolyzer through a feed inlet provision provided in the top lid of the pyrolyzer. The catalyst and feed plastics were added layer by layer and then mixed for the homogenous spread of the catalyst. The pyrolyzer unit was surrounded by a stainless steel outer chamber insulated with glass wool on the outside. The heating rate of the pyrolysis system was 10 °C min^−1^ till the set steady final temperature was reached. The temperature measurement was measured by a thermocouple, and the accuracy of the measurement was ±3 °C.^39^ The entire system was purged with nitrogen to maintain inert conditions. The catalytic pyrolysis experiments were carried out with different catalysts as shown in Table 1.^40,41^ Batch pyrolysis trials were conducted with different types of polyolefinic and multilayer packaging waste (LDPE, HDPE, PP, mixed plastic waste, and combination of MLPs) and catalysts (zeolite, ZSM-5, mordenite, and gamma aluminium). The feed to catalyst (10 : 1) ratio was used for all the experiments with a retention time of 90 min and a temperature range of 450–500 °C.^33,34^ The resultant product yield (oil, char, and gas) in wt% was quantified gravimetrically at the end of each experiment. The chemical characterization of the oil products was analyzed by GC-MS (Gas Chromatography-Mass Spectrometry).

**Table 1:** Types of plastic, feed ratio, and choice of catalyst in pyrolysis experiment

S. no.	Type of plastic waste	Plastic weight (g)	Feed ratio	Catalyst	Retention time (min)	Temperature (°C)
1	LDPE	1000	100	No catalyst	90	450–500
2	LDPE	1000	100	Zeolite	90	450–500
3	LDPE	1000	100	ZSM-5 (Com)	90	450–500
4	LDPE	1000	100	ZSM-5 (Syn)	90	450–500
5	LDPE	1000	100	Mordenite	90	450–500
6	LDPE	1000	100	Gamma alumina	90	450–500
7	HDPE	1000	100	Zeolite	90	450–500
8	PP	1000	100	Zeolite	90	450–500
9	Mixed plastic waste	1000	100	Zeolite	90	450–500
10	Metallized recycle plastic	1000	100	Zeolite	90	450–500
11	PET/MET/PET + polyolefinic mixed plastic waste	1000	50 : 50	Zeolite	90	450–500
12	BOPP/METBOPP	1000	50 : 50	Zeolite	90	450–500
13	BOPP/METBOPP + mixed plastic waste	1000	40 : 60	Zeolite	90	450–500
14	PET/FOIL/PET	1000	100	Zeolite	90	450–500

### 2.5. Chemical fingerprinting of pyrolysis oil

Chemical fingerprinting of oil and char was done through GC-MS (Agilent Technologies, USA). An HP-5 (30 m *×* 0.25 mm) silica-based cross-linked column was used. The injector and detector temperatures were set at 300 °C. The temperature was increased from 50 °C to 100 °C with a ramp rate of 10 °C min^−1^. The temperature was held at 100 °C for 120 s and was consequently increased to 250 °C by heating the system at a rate of 5 °C min^−1^. After holding the temperature at 250 °C for 2 min, the temperature was elevated to 300 °C with a ramp rate of 3 °C min^−1^ and was maintained at 300 °C for 15 min.^42^ A sample volume of 1 μL was injected in the splitless mode. Carrier gas (helium) was used at the rate of 1 mL min^−1^. MS was scanned from 35–550 amu at 1.562 u s^−1^. MS source and quadrupole temperatures were maintained at 150 °C and 230 °C, respectively. Calibration curves were prepared through multiple dilution 20 element multi-elemental total petroleum hydrocarbons (TPH) standard (Supelco, USA). Agilent Technologies Mass Hunter software was used to determine the amount of TPH present in the fuel oil. NIST mass spectral database was used to compare the mass spectra of the unknown organic compounds in the solvent extract.^32^

### 2.6. Reusability of catalyst

The leftover catalyst from the experiments was collected and reused to evaluate its effectiveness. Different combinations of used and unused catalysts were tried to study the optimal reuse of the catalyst in the pyrolysis process. The results indicated a reduction in oil yield on reusing the catalyst. The lower operating temperature of 450 °C decreased the stability and selectivity of the catalyst affecting oil yield as zeolite requires a higher reaction temperature.

### 2.7. Analysis of pyrolysis oil, gas, and char

The physicochemical characteristics of oil and char recovered from the polyolefinic and multilayer packaging-based waste were analyzed using ASTM methods. The properties of oil like gross calorific value, total sulfur content, kinematic viscosity, density, cetane index, carbon level, and flash point of fuel oil recovered from the plastic waste were characterized using ASTM methods (ASTM D4868-2017, ASTM D4868-2017, ASTM D4294-2016e1, ASTM D445-2017, ASTM D4052-2018, ASTM D976-2016, ASTM D92-2016B). The gross calorific value of the pyrolysis char was determined through a bomb calorimeter (ASTM D 5865:13). The concentration of heavy metals and sulfur present in the char were determined using ASTM D 6357:2011, ASTM E775 methods. The percentage of polycyclic aromatic hydrocarbons present in the char was determined through GC-MS.^16,43^

## 3. Results and discussion

### 3.1. Structural, textural, morphological, and acidic properties of ZSM-5 zeolite

**Fig. 2:**
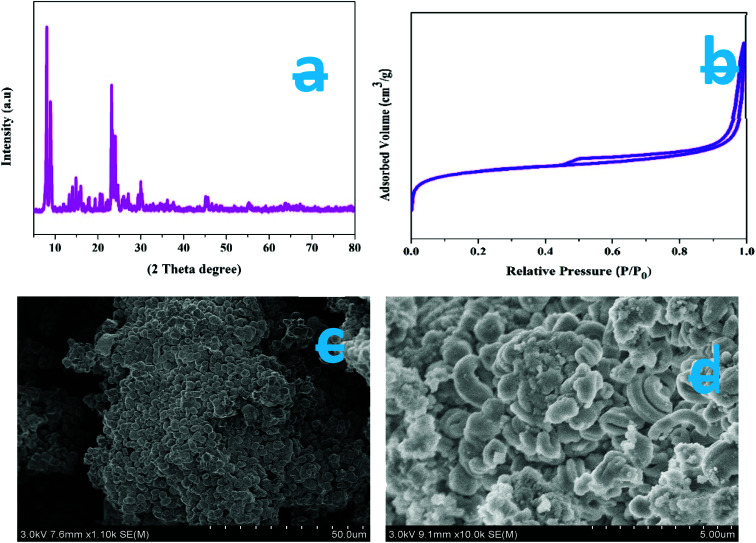
(a) XRD patterns of as-synthesized samples, (b) N_2_ adsorption/desorption isotherms of as-synthesized ZSM-5 zeolite, (c & d) HR-SEM images of synthesized ZSM-5 catalyst.

X-ray diffraction (XRD) was carried out at a wavelength of 1.542 Å (Cu Kα radiation) and was used to identify the crystalline phases present in the ZSM-5 zeolite catalyst. Fig. 2a shows the large-angle powder XRD patterns of the synthesized ZSM-5 zeolite prepared by the hydrothermal method. The observed characteristic diffraction peaks at *θ* = 7.98°, 8.82°, 14.82°, 23.14°, 23.96°, and 24.44° are associated with [011], [020], [031], [051], [303], and [313] planes with the *d*-spacing values of 1.11, 1.00, 0.59, 0.39, 0.37, and 0.36 nm, respectively. These values were completely intrinsic to the ZSM-5 zeolite catalyst structure and correspond to the JCPDS card no: 42-0024. The XRD results indicate that no diffraction peaks of the impurity phase were found, and the specific MFI structure was maintained on all the samples. The sharp peaks indicate that the synthesized ZSM-5 zeolite samples possess good crystallinity. The XRD result is similar to the one reported in the literature.^44^ The average crystallite size of the as-synthesized ZSM-5 zeolites is 30 nm and was calculated using the Debye–Scherrer formula *L* = 0.89*λ*/(*β* cos *θ*), where *L* is the average crystallite size, *λ* is the X-ray wavelength (0.154 nm), *θ* is the Bragg diffraction angle, and *β* is the full width at half maximum (FWHM) of the observed peak. The peak position and FWHM were obtained by fitting the peaks with two Gaussian curves in order to find the true peak position and width corresponding to the monochromatic CuKα radiation.

The nitrogen adsorption and desorption isotherms of the synthesized ZSM-5 zeolites are shown in Fig. 2b. The sample shows a type IV isotherm. The ZSM-5 sample exhibits a broader hysteresis loop from *P*/*P*_0_ = 0.45 to *P*/*P*_0_ = 1, which is due to the formation of additional mesopores. The experimentally determined surface area and pore volumes of synthesized ZSM-5 are 326.6 m^2^ g^−1^ and 0.38 cm^3^ g^−1^ (micropore volume, 0.08 cm^3^ g^−1^), respectively. The micropore volumes of the three samples are nearly the same. These results indicate that the additional mesoporosity was generated alongside the microporosity.

Fig. 2c and d are the HR-SEM images of synthesized ZSM-5, zeolites, respectively. The SEM images clearly suggest that all the samples obtained show a similar hexagonal cubic-like morphology and relatively have similar hexagonal cubic micro-blocks of about 320–360 nm in length without agglomeration. Furthermore, it is noted that the individual hexagonal cubic micro-blocks are made up of closely packed nanocrystals. The surface of ZSM-5 zeolite micro-blocks was slightly rougher. The rough surface is likely due to the hexagonal cubic micro-blocks that are constructed by the numerous small nanosized primary ZSM-5 particles, whose sizes vary from 26 to 35 nm, as implied by the XRD results. Also, from Fig. 2c and d, it is noticed that the nanosized primary ZSM-5 particles are aggregated with voids. A slight variation is noticed in the particle sizes measured by XRD and SEM. The small difference arises since SEM measurements are based on the difference between the visible grain boundaries, whereas XRD calculations measure the extended crystalline region that diffracts X-rays coherently. Hence, the XRD technique is relatively straightforward and accurate.

**Fig. 3:**
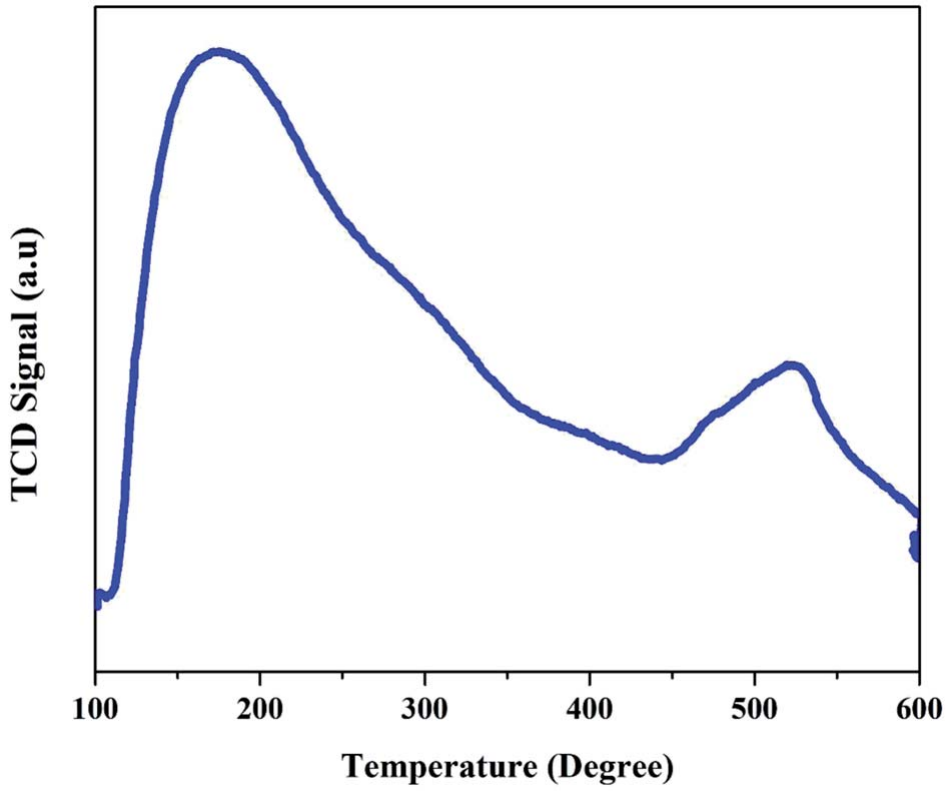
NH_3_-TPD profiles of as-synthesized ZSM-5 zeolite.

The acidic properties of the synthesized ZSM-5 zeolite catalysts were evaluated by the NH_3_-TPD technique. The corresponding TPD profiles of the ZSM-5 zeolites displayed in Fig. 3 show that ZSM-5 zeolite has two desorption peaks observed at around 200 °C (low-temperature peak) and 500 °C (high-temperature peak). The low-temperature peak is attributed to the ammonia desorption from weak acid sites, though the high-temperature peak is attributed to the ammonia desorption from strong acid sites (Brønsted and Lewis acid sites). The weak acid strength of ZSM-5 zeolite is due to the presence of amorphous aluminosilicates of the macropore walls.

### 3.2. Batch pyrolysis experiment with polyolefin and PSW plastic waste

#### 3.2.1 Comparison of thermal and catalytic pyrolysis

Pyrolysis temperature range of 450 to 500 °C was applied during the thermocatalytic depolymerization of different varieties of plastics. Zeolite beads were used as the catalyst material for the two-stage catalytic cracking of polymers. Zeolite was added into the pyrolyzer reactor along with the plastics for the *in situ* cracking process and the ratio of plastic and zeolite was fixed as 10 : 1, which was chosen based on previous studies. Petroleum hydrocarbons produced during pyrolysis were cracked in an *ex situ* manner. Pyrolysis product yield (oil, char, and gas) was estimated based on the weight obtained for the char and oil at the end of the pyrolysis process, and the pyrolysis product yields obtained at different conditions are given in Table 2. In the case of thermal pyrolysis (without catalyst), 37 wt% and 15 wt% of oil and char yields were obtained, respectively. In the case of catalytic pyrolysis, a maximum yield of 70 wt% of oil was observed for the same temperature range. The yield of char significantly reduced from 15% to 8% in the presence of the catalyst, similar to the drop in the yield of gaseous compounds (8%). The strong acidic properties and microporous crystalline structure of the catalyst enable increased cracking and isomerization, leading to an increased breakup of the larger molecules to smaller molecules forming gaseous and liquid yields. However, compared to other studies where the yield of gaseous compounds has increased in the presence of the catalyst, the present study observes an increase in the yield of oil. The increase in oil yield over the gaseous output compared to other studies could be influenced by several factors such as the size of the catalyst and the nature of contact between the catalyst and the feedstock, indicating the effect of conditions on the yield of both thermal and catalytic pyrolysis.

**Table 2:** Pyrolysis product yield (oil, char, and gas) at the end of the pyrolysis process

S. no.	Input quality	Catalyst	Temp set point (°C)	Product output (wt%)		
				Oil	Gas	Char
1	LDPE	Without catalyst	450–500	37	48	15
2	LDPE	Zeolite	450–500	50	42	8
3	LDPE	ZSM-5 (Syn)	450–500	70	16	14
4	LDPE	ZSM-5(Com)	450–500	46	12	42
5	LDPE	Mordenite	450–500	44	36	20
6	LDPE	Gamma alumina	450–500	40	32	28
7	HDPE	Zeolite	450–500	52	11	37
8	PP	Zeolite	450–500	32	31	37
9	Mixed plastic waste	Zeolite	450–500	53	35	12
10	Metallized recycle plastic	Zeolite	450–500	13	17	70
11	PET/MET/PET + polyolefinic mixed plastic waste	Zeolite	450–500	30	35	35
12	BOPP/METBOPP	Zeolite	450–500	60	25	15
13	BOPP/METBOPP + mixed plastic waste	Zeolite	450–500	35	30	35
14	PET/FOIL/PET	Zeolite	450–500	10	42	48

#### 3.2.2 Pyrolysis product yield

Pyrolysis of commercial LDPE plastic bags resulted in pyrolysis oil yield of 44–49 wt%, a gas yield of 41–50 wt%, and char yield of 5–9 wt% for *in situ* and/or *ex situ* catalytic pyrolysis process. Among which the zeolite catalyst provided in both *in situ* and *ex situ* arrangement resulted in the highest gas yield of 50 wt% and lowest solid yield of 8 wt% with the oil yield of 42 wt%. Various studies have also reported the increased production of gas with the utilization of zeolite on polyethylene compared to the gas yield of catalytic pyrolysis on other plastic feedstocks (using ZSM), reflecting the high acidic nature of catalyst incentivizing cracking of large molecules.^7,45^

Pyrolysis of waste plastics obtained from the municipality of Chennai resulted in generating 48–58 wt% of oil and 27–35 wt% of char. Similar to commercial plastic LDPE bags, municipal mixed plastics resulted in high gas yields for the zeolite provided in both *in situ* and *ex situ* ways. The addition of graphite to the mixture in the *ex situ* zeolite catalyst experiment resulted in the highest oil yield of 58.65 wt%, a gas yield of 14.35 wt%, and a char yield of 27 wt%. The increase in the yield of oil due to the addition of graphite could be attributed to the surface area and functional groups present in the graphite leading to enhanced cracking of the compounds.

Pyrolysis of HDPE waste produced char of 37 wt% and oil of 52 wt%. The reason for higher char yield was mainly due to higher inorganic content present in the plastics and unconverted plastics due to incomplete pyrolysis.^46^ Apparently, laminated metalized plastic resulted in the higher char production of 70 wt% and the least oil yield of 13 wt% due to higher aluminum and zinc metal concentrations and filler material present in the plastics. Commercial polypropylene plastic resulted in the production of 32 wt% oil, 31 wt% gas, and 37 wt% char.

**Fig. 4:**
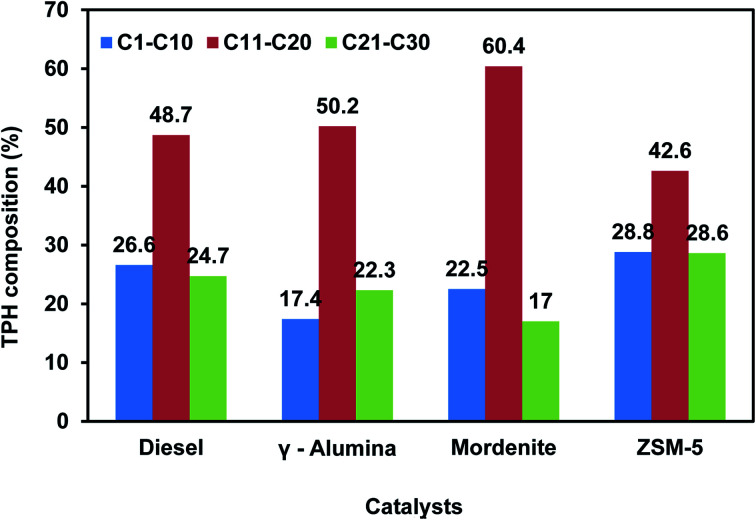
TPH distribution in LDPE pyrolysis oil with different catalysts.

Different types of plastic wastes like biaxial oriented polypropylene (BOPP), metalized biaxial oriented polypropylene layers (MET BOPP), polyethylene terephthalate (PET), metalized polyethylene terephthalate (MET/PET), polyethylene terephthalate combined polyethylene (PET/PE), and mixed plastic wastes were pyrolyzed in a batch pyrolysis system of 1 kg to determine the oil, gas, and char distributions. Various catalysts like zeolite, mordenite, ZSM-5, HZSM-5, and MCM-41 were extensively studied by researchers for polyolefin-based plastic wastes.^17,47^ BOPP/MET BOPP type plastic waste yielded 65–70% oil. BOPP/MET BOPP (40%) in combination with mixed plastic waste (60%) gave around 35–40% oil yield. PET/poly and mixed plastic waste yielded around 30–40% oil due to the presence of PE and PP present in the mixed plastic waste. PET/MET PET/POLY (50%) and mixed plastic waste (50%) yielded around 25–30% oil due to the presence of PE and PP present in the mixed plastic waste. The gas and char composition account for 70–75% of PET and MET/PET-based plastic waste. The presence of aluminium foil and PET in the plastic waste (PET/FOIL/PET) gives only 10–15% oil with more char. The details of plastic feed, catalyst, the temperature used, and product output obtained (wt%) in the pyrolysis experiments are listed in Table 2. The TPH composition (C1–C10, C11–C20, and C21–C30) of diesel was compared with TPH composition of pyrolysis oil obtained with catalysts (ZSM-5, mordenite, and γ-alumina) and displayed in Fig. 4. The TPH composition of pyrolysis obtained with ZSM-5 catalyst showed an almost similar composition to diesel.^48^

### 3.3. Plastic pyrolysis oil and char characterization

#### 3.3.1. Analysis of pyrolysis oil

In this study, we tried to evaluate the performance of zeolite with different layers of polyolefin waste and PSW combinations present in flexible packaging and municipal solid waste. The major steps involved in the catalytic reactions during pyrolysis are cracking, oligomerization, cyclic aromatic compound formation, and isomerization reactions.^47^ The secondary reactions are mainly based on cracking, rupture of rings, and aromatization, which lead to the formation of hydrocarbons in the range of (nC4 to nC10) and low molecular weight aromatics.^49^ The formation mechanism of linear alkenes can be generally attributed to mid-chain b-scission reactions of midchain secondary alkyl radicals. Analysis of pyrolysis oil through GC-MS shows significant production of a large fraction of unsaturated hydrocarbons that include linear alkanes and alkenes (dienes, trienes) in the pyrolysis oil.^50^ The GC-MS chromatogram of pyrolysis oil is shown in ESI Fig. S1.†

**Fig. 5:**
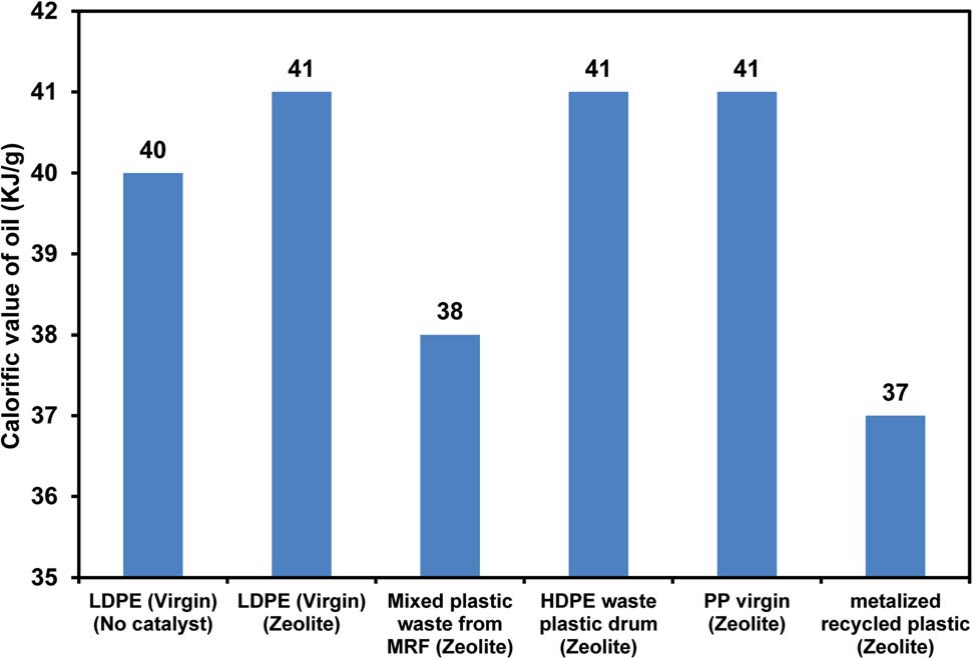
Variation in TPH distribution for waste plastic with different compositions.

The physicochemical and thermal characteristics of pyrolysis oil obtained from LDPE compared with commercial diesel and fuel oil are given in Table 3. Calorific value is one of the important characteristics based on which the quality of the fuel is evaluated for further applications. The calorific value for various plastics with zeolite catalysts is displayed in Fig. 5. The calorific values of commercial-grade LDPE were within the range of 40 to 42 kJ g^−1^. The calorific values for mixed waste plastics obtained from the municipal corporation were found to be 38 to 39 kJ g^−1^. The calorific values of HDPE waste plastic and laminated metalized plastic-based pyrolysis oils were observed to be 41 and 37 kJ g^−1^, respectively. The gross calorific value of the pyrolysis oil obtained from different combinations of PSW varied between 45–30 kJ g^−1^. BOPP-based plastic waste gave higher oil yield and calorific value compared to PET-based PSWs. The calorific value of mixed waste plastics obtained from the municipal corporation with PSWs like BOPP and PET was found to be around 45 and 30 kJ g^−1^. The calorific values of BOPP and laminated metalized plastic-based pyrolysis oil were observed to be 45 and 30 kJ g^−1^, respectively. These values are found to be similar to those reported for waste plastic pyrolysis oil in other studies, which are in the range of 30–47 kJ g^−1^ depending on the input plastic quality.^51,52^ The lowest calorific value of 30 kJ g^−1^ obtained from PET samples can be due to terephthalate formation during the thermal degradation process. Based on the results, it is evident that the pyrolysis oil obtained from the processing of PSWs with mixed plastic waste will be a feasible option for further applications that require a certain calorific value of the oil.

**Table 3:** Properties of the pyrolysis oil from different types of plastics

S. no.	Pyrolysis oil	Kinematic viscosity at 40 °C (cSt)	Gross calorific value (kJ g^−1^)	Sulfur content (wt%)	Density at 15 °C kg m^−3^	Flash point (°C)
1	LDPE with ZSM-5	11.7	41	<0.1	780	31
2	LDPE without catalyst	1.08	40	∼0.0017	811.9	79
3	LDPE with zeolite	2.49	42	∼0.009	814.7	52
4	Commercial diesel	1–4.11	45	<0.1–0.6	799	52–96
5	Commercial foil-2	2–3.6	40–45	0.1–0.6	900–1010	40–75

#### 3.3.2. Effect of plastic-type on carbon number distribution in the pyrolysis oil

The hydrocarbon range present in different plastic pyrolysis oils was compared with diesel to evaluate the combustion performance. The C5–C10, C11–C20, and C21–C30 carbon numbers present in BOPP, METBOPP, PET/FOIL/PET, PET/MET PET/PE, and BOPP/MET PET samples were analyzed from GC-MS chromatogram. The percentage area of (C5–C10) compounds varies in the range of 8.9–23.6 (%). The medium-range hydrocarbons (C11–C20) vary from 27.5–60.5(%). The heavier fractions (C21–C30) vary in the range of 24.7–55.7 (%). Pyrolysis oil obtained from polyethylene have a higher calorific value due to the presence of paraffinic, olefinic, and aromatic hydrocarbons [C12, C14, C21]. Long-chain hydrocarbons generally have low combustion temperatures.

#### 3.3.3. Pyrolysis char characterization

The pyrolysis char obtained from different batch experiments varied in the range of 8–70 (%) depending upon the type of plastic used. Char yield from polyolefin-based plastics LDPE, PP, and BOPP varied in the range of 8–20 (%). The additives added in the HDPE-based waste increased the char quantity to 30(%). PSW based waste yielded 15–48 (%) char. PET/FOIL/PET combination and laminated metalized recycled plastic gave a maximum amount of char in the range of 50–70 (%). The char obtained from plastic pyrolysis using different plastic wastes like LDPE, biaxial oriented polypropylene (BOPP), and PET/PET-based char were characterized for the presence of heavy metals, and concentrations (mg kg^−1^) in the range of *viz.*, chromium (15.36–97.48), aluminium (1.03–2.54), cobalt (1.0–5.85), copper (115.37–213.59), lead (89.12–217.3), and nickel (21.05–175.41), respectively, were found. Sulfur as (SO_3_) concentrations in the char varied from 34–441 mg kg^−1^. The composition of aliphatic, aromatic, and polar fractions of char was analyzed through GC-MS. The peaks were identified by comparing them with the NIST library. The compounds having a carbon number ranging from nC13 to nC33 showed an aliphatic fraction. The retention time of the aliphatic fraction showed a hump representing the unresolved hydrocarbon fraction.^53^

## 4. Conclusion

The study aimed to develop a low-cost catalyst and conduct experiments with a larger quantity of feed to obtain products such as oil, char, and non-condensable gas. A ZSM-5 zeolite catalyst was synthesized by hydrothermal method using metakaolin as an alumina source. The XRD analysis confirmed the formation of ZSM-5 phases with a high surface area of 326.6 m^2^ g^−1^ (BET analysis) and hexagonal cubic-like morphology (SEM analysis). From TPD analysis, it was confirmed that the synthesized catalysts possess both Brønsted and Lewis acid sites. Catalytic pyrolysis of mixed plastic waste to fuel oil experiment was tested with ZSM-5 zeolite (commercial and synthesized) catalysts along with other catalysts. In the case of thermal pyrolysis (without catalyst), 37 wt% and 15 wt% of oil and char yields were obtained, respectively. Among the tested catalysts, the synthesized ZSM-5 zeolite produced high oil content (70 wt%) with low char products (14 wt%) compared to other catalysts using LDPE as feed. Compared to polyolefin-based waste plastics, pyrolysis oil derived from PET-based MLPs and laminated metalized recycled plastics with mixed plastic yields less oil. The produced oil and char were characterized using GC-MS to determine the carbon range fraction in the oil, and other instruments were used to measure the physico-chemical properties of these products. The pyrolysis oil produced from LDPE by ZSM-5 catalyst possessed a gross calorific value of 41 kJ g^−1^, almost equal to commercial diesel and fuel oil.

## Conflicts of interest

There are no conflicts to declare.

## Supplementary Material

RA-012-D1RA08673A-s001
