# Supplementary material for: Conversion of plastic waste into fuel oil using zeolite catalysts in a bench-scale pyrolysis reactor
Source: RSC Adv. 2022 Mar 8;12(13):7612–20. doi: 10.1039/d1ra08673a (PMC8982165; doi:10.1039/d1ra08673a)
Supplement: RA-012-D1RA08673A-s001 [file RA-012-D1RA08673A-s001.pdf]

Electronic Supplementary Material (ESI) for RSC Advances.
This journal is © The Royal Society of Chemistry 2022

Supporting Information

Conversion of plastic waste into fuel oil using zeolite catalysts in a bench scale pyrolysis reactor

Krishnasamy Sivagami[1,4], Keshav V Kumar [3], Perumal Tamizhdurai[2*],

Dhivakar Govindarajan[1], Madhiyazhagan Kumar [3], Indumathi Nambi[*1,3]

[1]Environmental and Water Resources Division, Department of Civil Engineering, Indian Institute of Technology Madras, Chennai- 600 036

[2]Department of Chemistry, Dwaraka Doss Goverdhan Doss Vaishnav College (Autonomous), E.V.R. Periyar Road, Arumbakkam, Chennai, Tamil Nadu 600 106, India

[3]Samudhyoga Waste Chakra Private Limited, IIT Madras Research Park, Tharamani, Chennai – 600 113, India.

[4] Industrial Ecology Group, School of Chemical Engineering, Vellore Institute of Technology, Vellore – 632 014, Tamil Nadu, India

[*]**Corresponding author**:

Dr.P.Tamizhdurai: Tel: +91-9677146579 Email: **p.tamizhdurai@dgvaishnavcollege.edu.in**

Dr. Indumathi M. Nambi Tel: +91-44-2257 4289 Email: **indunambi23@iitm.ac.in**

Fig. S1. GC-MS Chromatogram of different pyrolysis oil.

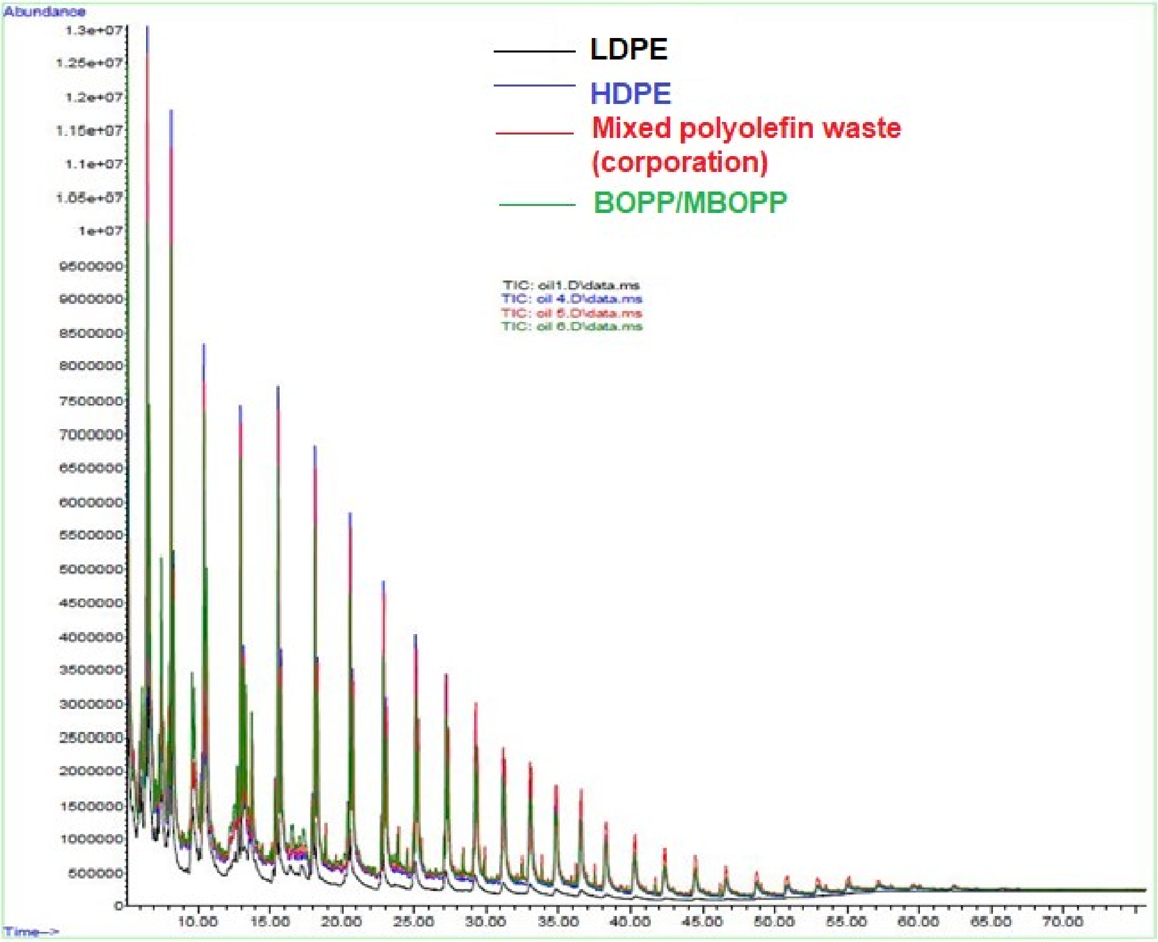

Figure S1
